# Introducing Berry phase gradients along the optical path via propagation-dependent polarization transformations

**DOI:** 10.1515/nanoph-2021-0560

**Published:** 2021-11-19

**Authors:** Ahmed H. Dorrah, Michele Tamagnone, Noah A. Rubin, Aun Zaidi, Federico Capasso

**Affiliations:** Harvard John A. Paulson School of Engineering and Applied Sciences, Harvard University, Cambridge 02138, MA, USA; Fondazione Istituto Italiano di Tecnologia, Genova, Italy

**Keywords:** metasurfaces, nanophotonics, nondiffracting beams, optical angular momentum, polarization optics, structured light

## Abstract

As a classical or quantum system undergoes a cyclic evolution governed by slow change in its parameter space, it acquires a topological phase factor known as the *geometric* or *Berry* phase. One popular manifestation of this phenomenon is the Gouy phase which arises when the radius of curvature of the wavefront changes adiabatically in a cyclic manner, for e.g., when focused by a lens. Here, we report on a new manifestation of the Berry phase in 3D structured light which arises when its polarization state adiabatically evolves along the optical path. We show that such a peculiar evolution of angular momentum, which occurs under free space propagation, is accompanied by an accumulated phase shift that elegantly coincides with Berry’s prediction. Unlike the conventional dynamic phase, which accumulates monotonically with propagation, the Berry phase observed here can be engineered on demand, thereby enabling new possibilities; such as spin-dependent spatial frequency shifts, and modified phase matching in resonators and nonlinear interactions. Our findings expand the laws of wave propagation and can be applied in optics and beyond.

## 1 Introduction

One of the many wonders of the quantum world manifests when a charged particle passes around a long solenoid; although the magnetic field is negligible in the region through which the particle passes (outside the solenoid) and the particle’s wavefunction is negligible inside the solenoid, nevertheless the particle’s wavefunction still experiences a phase shift as a result of the enclosed magnetic field [1]. This mysterious interaction—confirmed by various experimental setups [2–10]—is known as the Aharonov–Bohm effect and highlights the role of electromagnetic potentials, *φ* and **A**, which were often debated as mere mathematical constructs, in enforcing the principle of locality [11]. Importantly, this phase shift is topological in nature; it does not depend on the shape of the path traversed by the particle but rather its *topological invariants*. As much as it is profound, however, this quantal phase accumulation descends from a more deeply rooted origin. Notably, in his 1984 seminal work [12], Sir Michael Berry showed that “A quantal system in one eigenstate, slowly transported around a circuit by varying the parameters in its Hamiltonian, will acquire a geometrical phase factor in addition to the familiar dynamical phase”. This additional phase factor is referred-to today as the *geometric* or *Berry* phase.

**Figure 1:**
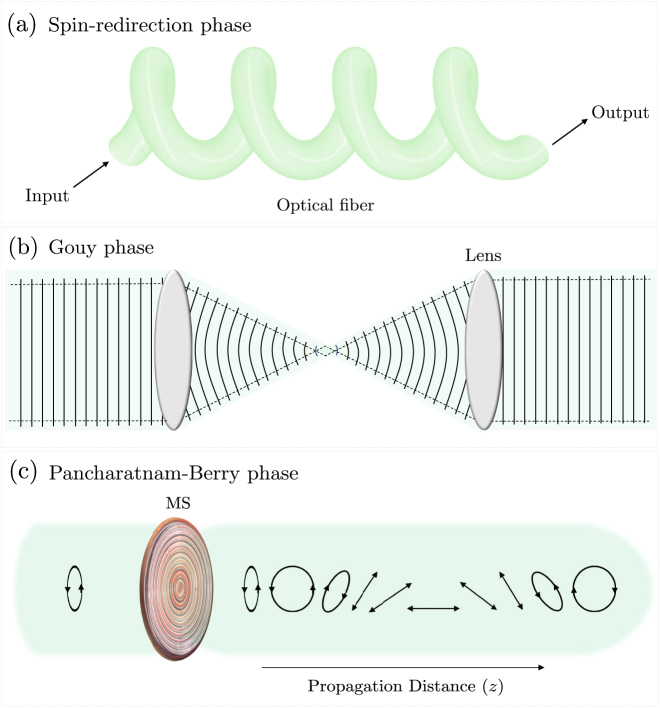
Examples of Berry phase manifestations in optics. (a) Spin-redirection phase arises when a photon is adiabatically transported in momentum space; a scenario widely encountered in helically wounded optical fibers in which the output mode experiences a topological phase shift (independent from its dynamic phase) as it traces a helical trajectory in space. (b) Gouy phase arises upon focusing as the radius of curvature of the wavefront is slowly cycled in its parameter space. (c) A new manifestation of the Berry phase appears in a class of polarization meta-optics which adiabatically transforms the polarization state, locally, along the optical path. The accumulated phase is topological and can be engineered on demand by designing the output polarization response.

The geometric phase is of a fundamental significance as it underpins many physical phenomena [13, 14]. For instance, its classical analog explains the angular displacement observed in the Foucault pendulum [15]—known as the Hannay angle [16], it underlies the Zak phase encountered by Bloch electrons in 1D periodic lattices [17], and manifests in spin-dependent deformations of optical fields like, for e.g., spin–orbit coupling [18]. In optics, the two main classes of geometric phase are [13, 19]: (a) the spin-redirection geometric phase, and (b) the Pancharatnam–Berry phase [20]. The former arises when light with fixed polarization (or more generally; angular momentum) changes its direction in space—a situation encountered in helically wounded optical fibers [21–23] (Figure 1(a))—whereas the latter is typically observed when successively projecting light’s polarization in a cyclic manner using birefringent elements. For example, when light passes through a sequence of polarizing elements, causing its original state of polarization to traverse a cyclic trajectory on the Poincaré sphere, the output beam gains an additional phase shift governed by the topology of the path traversed in polarization space. The curvature of the Poincaré sphere, which visualizes all possible states of polarization, allows this phase factor to be geometrically evaluated as half the solid angle enclosed by the traversed topological path. Generalizations of this rule that apply to nonadiabatic and/or noncyclic topological deformations have also been reported [24, 25]. Notably, the well-known Gouy phase which accompanies a Gaussian beam as it changes its waist size under focusing also has deep connection with the Berry phase (Figure 1(b)). This additional phase factor arises as the complex radius of curvature of the Gaussian beam is adiabatically cycled in its parameter space, introducing a spread in the transverse momentum and thus a perturbation to the axial propagation constant, which can also be reconciled from the position–momentum uncertainty principle [26, 27]. Other manifestations of the geometric phase in optics are evident in the spin Hall effect of light [28–30], spin–orbit conversion of circularly polarized Gaussian beams via strong focusing [31, 32] or using metasurfaces with locally varying anisotropy [33–35], as well as temporal beating of polychromatic polarized light [36], and more recently, in Young’s double slit experiment invoking polarized vector fields [37]. In addition, higher order manifestations of the geometric phase exist in beams carrying orbital angular momentum [38–41]. Besides its scientific significance, the geometric phase plays a key role in many applications: from precision metrology [42], to high resolution microscopy [43], optical micromanipulation [31, 44, 45], and polarimetry [46, 47].

With recent advances in wavefront shaping, enabled by digital holography [48] and metasurfaces [49, 50], it became possible to sculpt light into complex topologies by structuring all its degrees-of-freedom, point-by-point, at the subwavelength scale, thus enabling new behaviors. For instance, a new class of meta-optics can now perform successive polarization transformations along the optical path, after a single interaction with incident light, thereby modifying its polarization at each plane thereafter [51]. Light topologies of this nature should in principle incur new physical dynamics connected with the geometric phase, as their spin angular momentum traces some path on the Poincarè sphere with propagation. Hence, the propagation dynamics of such beams cannot be solely described by the dynamic phase. Here, we explore this further; using a metasurface with shape birefringent unit cells, we sculpt the amplitude, phase, and polarization of incident light, point-by-point, transforming it into a quasi diffraction-less pencil-like beam. Importantly, we allow the spin angular momentum (polarization) of such a beam to evolve, in an adiabatic manner, as a function of the propagation distance (Figure 1(c)). We show that such a peculiar evolution in the angular momentum is accompanied by an additional phase shift which satisfies the criteria of the Pancharatnam–Berry phase and that is different from the familiar dynamic phase accumulated with propagation. Notably, the sign and accumulation rate of this geometric phase factor can be tailored on demand by judiciously designing the polarization transformation carried by the meta-optic. With this degree-of-freedom, one can design the phase gradient along the optical path, leading to new physical behaviors such as shifting the spatial frequency of the beam depending on its input polarization—a consequence of its energy–momentum conservation. In the following, we first revisit the design strategy of our recently polarization meta-optics then examine their underlying geometric phase factor and its physical consequences.

## 2 Longitudinally variable polarization meta-optics

### 2.1 Design strategy

Our goal is to construct vector beams with propagation-dependent spin angular momentum and then examine the evolution of their geometric phase. Bound by angular momentum conservation laws [52], however, this behavior can be realized only locally; i.e., the global angular momentum across the transverse section of the beam should always be conserved. Here, we adopt the design strategy first introduced in Ref. [51]. Consider a discrete superposition of forward propagating modes with different polarization states and propagation constants (wave vectors). Due to the constructive and destructive interference among these co-propagating modes, the polarization of the resulting waveform will be modulated with propagation in space. By properly selecting the weight (amplitude and phase) and polarization state of each mode, the polarization state of the envelope can be precisely controlled along the direction of propagation—see for e.g., Ref. [53] and review article [54]. We take a step further and assign 2-by-2 Jones matrices as the weighting coefficients for each forward propagating mode [51, 55]. Consequently, the resulting superposition will mathematically take the form of a 2-by-2 Jones matrix whose 4 elements undergo modulation in space. In essence, this propagation-dependent Jones matrix describes a polarization optic whose eigenvalues (retardance) and eigen vectors (fast axis orientation) changes in space. Light incident on such a device will modify its polarization state along the direction of propagation. We chose the Bessel profile as our forward propagating modes; hence our device implements the superposition

(1) $${\tilde{\psi }}^{\ell }\left(\rho ,\phi ,z,t\right)={\text{e}}^{-\text{i}\omega t}\overset{N}{\underset{m=-N}{\sum }}{\tilde{A}}^{\ell ,m}J_{\ell }\left(k_{\rho }^{\ell ,m}\rho \right){\text{e}}^{\text{i}\ell \phi }{\text{e}}^{\text{i}k_{z}^{\ell ,m}z}.$$

The term $J_{\ell }\left(k_{\rho }^{\ell ,m}\rho \right){\text{e}}^{\text{i}\ell \phi }{\text{e}}^{\text{i}k_{z}^{\ell ,m}z}$ denotes a Bessel mode of order *ℓ* propagating along the *z*-direction, whereas $k_{\rho }^{\ell ,m}$ and $k_{z}^{\ell ,m}$ are the transverse and longitudinal wavevectors, respectively, ${\left(k_{\rho }^{\ell ,m}\right)}^{2}+{\left(k_{z}^{\ell ,m}\right)}^{2}={\left(\omega /c\right)}^{2}$ , and ${\tilde{A}}^{\ell ,m}$ are 2-by-2 Jones matrices, representing the coefficients of each Bessel mode in the series. Here, *ρ* and *ϕ* are the radial and azimuthal coordinates, respectively. The choice of Bessel beams as the forward propagating modes is not fundamental but rather advantageous, owing to their nondiffracting and self-healing characteristics [56]. The profile ${\tilde{\psi }}^{\ell }\left(\rho ,\phi ,z=0,t=0\right)$ —to be implemented via a metasurface—represents a spatially varying 2D distribution of Jones matrices that can, point-by-point, act on incident Jones vectors, i.e. polarized light. Notably, a target polarization response described by the “*z*-dependent” Jones matrix, which we dub ${\tilde{F}}^{\ell }$ , can be imparted along the optical path of the beam, over the longitudinal distance *L*, provided that its coefficients ${\tilde{A}}^{\ell ,m}$ satisfy the Fourier relation [51]

(2) $${\tilde{A}}^{\ell ,m}=\frac{1}{L}\overset{L}{\underset{0}{\int }}{\tilde{F}}^{\ell }\left(z\right){\text{e}}^{-\text{i}\frac{2\pi }{L}mz}\;dz.$$

The matrix-valued coefficients ${\tilde{A}}^{\ell ,m}$ are substituted in Eq. (1) to evaluate the Fourier series ${\tilde{\psi }}^{\ell }$ which locally approximates the desired *z*-dependent polarization response, ${\tilde{F}}^{\ell }$ . In essence, a wavefront shaping medium that takes the form ${\tilde{\psi }}^{\ell }\left(x,y,z=0\right)$ at an initial *z*-plane, transverse to the longitudinal direction, will interact with incident light (only once) leading to a transformation in its state of polarization at each consecutive *z*-plane thereafter. This mimics a scenario where light exiting from the device encounters several virtual retarders or polarizers—each with a slightly rotated principle axis—placed along the optical path, as described by ${\tilde{F}}^{\ell }$ . Notably, the evolution in the polarization state of the resulting envelope occurs only locally at the beam’s center.

**Figure 2:**
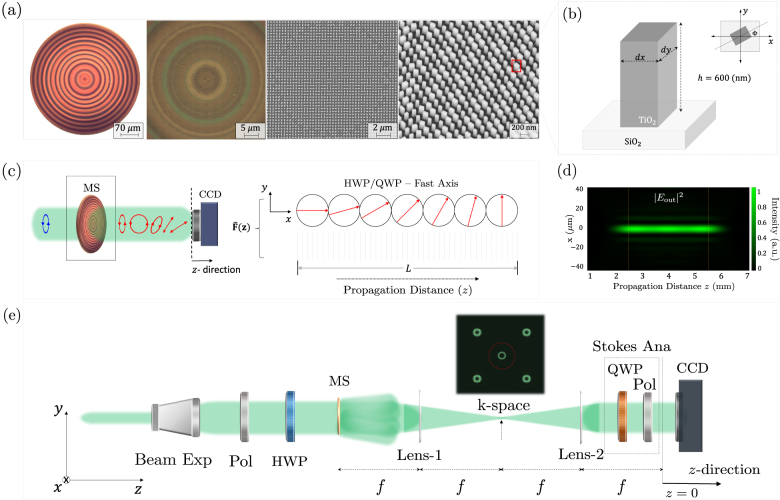
Longitudinally variable polarization optics. (a) Optical micrographs and SEM images of a meta-optic which performs variable polarization transformations along the optical path. (b) Each metasurface unit cell is composed of a rectangular nanofin whose transverse dimensions and angular orientation varies, point-by-point, behaving locally as a waveplate with variable retardance and fast axis orientation. Here, the nanofins are made of titanium dioxide (TiO_2_) with a fixed height of 600 nm on top of a glass substrate. (c) The polarizing meta-optic generates a waveform that changes its polarization along the direction of propagation; as if interacting with virtual retarders of different fast axis orientation, located along *z*. The target polarization response, ${\tilde{F}}^{\ell }$ , can be chosen to mimic a waveplate, for e.g., a half-wave plate (HWP) or quarter-wave plate (QWP), that adiabatically rotates its orientation by an angle *θ* with the *x*-axis, as a function of *z*, as depicted by the red arrows. This target response is realized over the finite space region *L*. (d) Simulated intensity profile of the output waveform; a quasi nondiffracting pencil-like beam that changes its polarization with propagation. (e) Experimental setup used to characterize our polarizing devices. A 532 nm coherent Gaussian beam is expanded and collimated and then passes through a polarizer (Pol) and half-wave plate (HWP) to modify the input polarization state. The output response of the metasurface (MS) is filtered and imaged with a 4-*f* system onto a CCD camera mounted on a translational stage in the *z*-direction. Here, Lens-1 performs a Fourier transform allowing the desired spectrum to be filtered (in the far field) from higher orders, and Lens-2 brings the filtered pattern back to real space. To measure the output polarization at each *z*-plane, Stokes polarimetry has been performed using a quarter-wave plate (QWP) and a polarizer.

To realize the transverse profile ${\tilde{\psi }}^{\ell }\left(x,y,z=0\right)$ , one needs an implementation medium that locally takes the functional form of 2-by-2 Jones matrices. To achieve this we deploy metasurfaces [50, 57] composed of nano-structured rectangular waveguides with shape birefringence. The dimensions and angular orientation of these birefringent nanofins can be chosen, point-by-point on the metasurface, to approximate the target profile $\tilde{\psi }\left(x,y,z=0\right)$ as fully detailed in Supplementary Note 1 and Ref. [51]. The metasurfaces were fabricated using electron beam lithography and atom layer deposition following the procedure in Ref. [58]. Figure 2(a) shows optical and SEM images of a meta-optic (0.462 mm in diameter) which performs variable polarization transformations along the optical path. At the macroscopic level, the profile consists of concentric rings; consistent with the Bessel profile of Eq. (1) which produces diffraction-less pencil-like beams at the output, as shown in Figure 2(d). At the nanoscale, each rectangular nanofin, schematically shown in Figure 2(b) behaves as a waveplate that modifies the polarization state of incident light, point-by-point, owing to its shape birefringence. At any propagation distance, *z*, the polarization state of the envelope (on-axis) is given by $\left|E_{\text{out}}\left(\rho =0,z\right)\rangle =\tilde{F}(z)|E_{\text{inc}}\right\rangle$ ; as if input light witnesses a series of polarization optics, with rotated birefringence axes, along *z*. This is illustrated in Figure 2(c) where we set $\tilde{F}(z)$ to take the form of longitudinally variable retarders, adiabatically rotating their fast axis orientation (*θ*) from 0° to 90°, with respect to the horizontal axis, as a function of *z*.

### 2.2 Results

**Figure 3:**
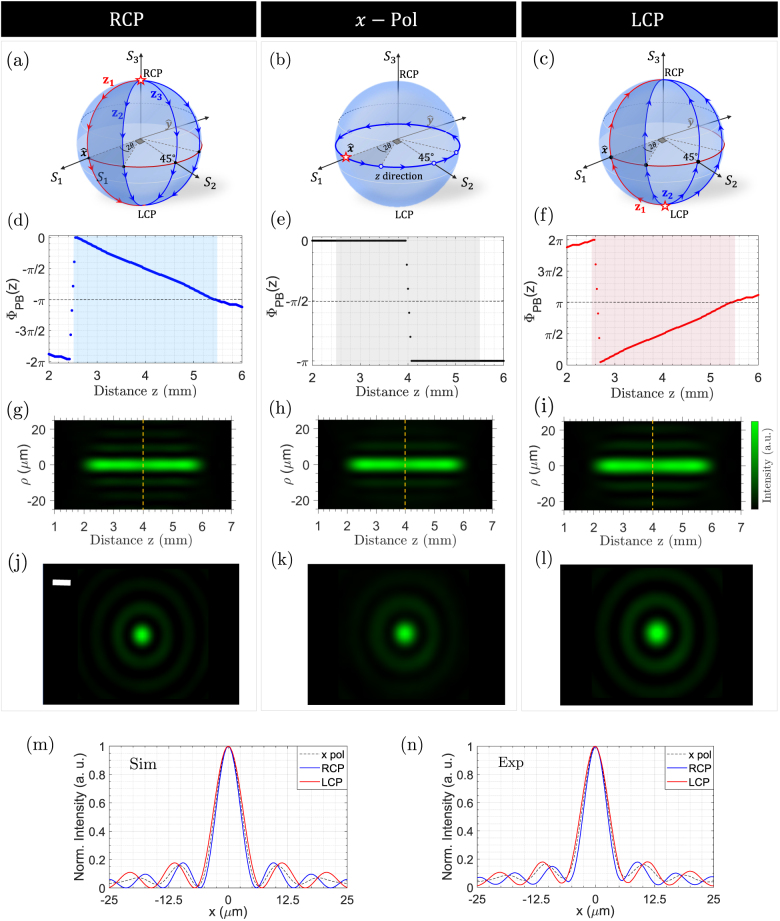
Spatial evolution of polarization and Pancharatnam–Berry phase in longitudinally varying meta-optics. (a–c) Trajectory of the polarization state, visualized on the Poincaré sphere, when the meta-optic is illuminated by RCP (a), linear ( $\hat{x}$ ) (b), and LCP polarized light (c). Here, the meta-optic mimics an HWP which rotates its fast axis orientation by an angle *θ*, which varies as function of *z*. (d–f) Geometric phase associated with each of the propagation-dependent polarization transformations in (a–c). (g–i) Longitudinal intensity profile at the output of the meta-optic under each incident polarization. The dashed lines mark cross sectional cuts at *z* = 4 mm. The corresponding transverse profiles are depicted in (j–l) and show a slight variation in the beam’s size, depending on the input polarization. The scale bar is 10 μm. (m–n) Simulated and measured 1D cuts, from the transverse profiles in (j–l), exhibiting the polarization-dependent shift in the beam size—i.e., its spatial frequency perturbation.

We consider a polarization meta-optic whose response $\tilde{F}(z)$ mimics that of a half-wave plate with a fast axis orientation that adiabatically rotates from 0° to 90°, with respect to the horizontal axis, as a function of *z*. The output response of this device has been fully characterized using a 4-*f* optical system under different input polarizations as shown in Figure 2(e). In each case, the output beam was detected by a CCD after passing through a combination of polarization optics which analyze for $\hat{x}$ , $\hat{y}$ , 45°, and circular polarization states, to measure the output polarization state via Stokes polarimetry [59]. When illuminated by linearly polarized light, this device will rotate the polarization of the output beam, continuously, as it propagates along the *z*-direction. In this case, the polarization state will evolve on the equator of the Poincaré sphere, returning to the initial state, as depicted in Figure 3(b). Circularly polarized light incident on the same device, on the other hand, will reverse its handedness, transitioning from the north to the south pole of the Poincaré sphere, or vice versa, as shown by the arrows in Figure 3(a) and (c). This transition can take one of many possible paths depending on the orientation of the device’s fast axis, which varies along *z*, as highlighted in the same figure.

As the beam propagates in space it acquires a monotonically increasing dynamic phase. Additionally, the evolution of the beam’s state of polarization with propagation gives rise to another phase factor, modifying the overall phase. To illustrate this, we examine the phase acquired by vector beams with spatially evolving polarization in comparison to a reference beam, with fixed polarization state, propagating for the same distance. In this case, the difference between the propagation phases accumulated by each beam will vanish, thus any relative phase shift will be attributed to the polarization transformation—i.e., a Pancharatnam–Berry phase arising only in one of the two beams. More specifically, we consider the output response of our meta-optic under the three input polarization states: RCP, LCP and *x*-polarized light. We compare this response to that of a reference device in which $\tilde{F}(z)$ takes the form of HWP whose fast axis does not rotate along *z*. The former device generates a pencil-like beam with spatially evolving polarization whereas the latter produces a beam whose polarization state is fixed. At a given propagation distance, the relative phase shift between the two output beams is a manifestation of the Pancharatnam–Berry phase.

Here, we adopted Pancharatnam’s operational definition [20] which implies that the relative phase shift between two beams of different polarizations is the phase retardation which allows the intensity resulting from their mutual interference to be maximized. Figure 3(d)–(f) show the predicted relative phase shift considering the three input polarizations above. Note that circularly polarized light incident on our meta-optic (i.e., *z*-dependent HWP) would reverse its chirality at the output while gradually accumulating a phase that is negative (for RCP light) or positive (for LCP) as it propagates away from the device. At each *z*-plane, the final polarization state is the same but the path taken on the Poincaré sphere from the initial to the final state is different. The red and blue trajectories on the Poincaré sphere depict the responses of the *z*-dependent meta-optic and the reference device, respectively, where both trajectories coincide at the initial *z*-plane. The geometric phase is equal to half the solid angle enclosed by the two paths. In contrast, linearly polarized light will adiabatically rotate, evolving along the equator of the Poincaré sphere, only encountering a constant (*π*) phase shift, relative to the reference beam, after crossing the diametrically opposite point on the Poincare’ sphere.

### 2.3 Significance

Unlike conventional dynamic phase accumulated with propagation, the geometric phase shift observed here can be controlled along the direction of propagation, on demand, by choosing the polarization response, $\tilde{F}(z)$ . This polarization transformation provides direct route to engineering the gradient of the geometric phase along the optical path, which manifests as a shift in the spatial frequency of the output waveform. To reconcile this, recall from Eq. (1) that *ψ* ^*ℓ*^ is composed of 2*N* + 1 Bessel beams which are equally separated in the *k*_*z*_ space and centered at the longitudinal wavevector $k_{z}^{\ell ,0}$ . In the paraxial regime (*k*_*z*_ ≫ *k*_*ρ*_), the ensemble *ψ* ^*ℓ*^ accumulates a propagation phase $∼{\text{e}}^{\text{i}k_{z}^{\ell ,0}z}$ . After including the additional Berry phase factor, which varies with *z*, the phase of the ensemble becomes $∼{\text{e}}^{\text{i}\left(k_{z}^{\ell ,0}z+\Phi_{\text{PB}}\left(z\right)\right)}$ , where Φ_PB_ is the additional Pancharatnam–Berry phase. This modification in the overall phase term can also be expressed as $∼{\text{e}}^{\text{i}\left(k_{z}^{\ell ,0}z+\int k_{\text{PB}}\left(z\right)dz\right)}$ where *k*_PB_ is *∂*Φ_PB_/*∂z* and denotes a perturbation to the original wave vector, $k_{z}^{\ell ,0}$ . From the consistency relation, $k_{\rho }^{2}+k_{z}^{2}={\left(\omega /c\right)}^{2}$ , a modification to the effective *k*_*z*_ shall also modify the transverse wavenumber, *k*_*ρ*_. This shift manifests as a change in the beam’s diameter and depends on the input polarization state of the beam, as depicted in Figure 3(g)–(i). Notice that the size of the beam’s central spot at the output is slightly smaller under RCP illumination compared to linearly polarized light. Here, |*k*_PB_| is estimated to be *π*/0.003 m^−1^, causing a shift in the order of ±3 × 10^4^ m^−1^ in the transverse spatial frequency. This is consistent with the negative phase gradient along *z*, i.e. its slope shown in Figure 3(d), which suggests a reduction in the *k*_*z*_ component of the wavevector. This phenomenon is spin-dependent; when the chirality of input light is reversed so that the same meta-optic is illuminated by LCP light, the effect is reciprocated and the beam size becomes larger, as seen in the transverse profiles of Figure 3(j)-(l). To better visualize the perturbation in the beam’s size, we plotted the simulated and measured 1D cuts of these transverse profiles in Figure 3(m) and (n) which not only show the variation in the spot size but also the spatial oscillation in its side lobes. The implication of these shifts in the *k*-vector is far-reaching as it fundamentally affects the momentum matching condition which is one of the pillars of Snell’s law [49], nonlinear optics, and mode selection in cavities.

## 3 Direct observation of spatially evolving geometric phase

**Figure 4:**
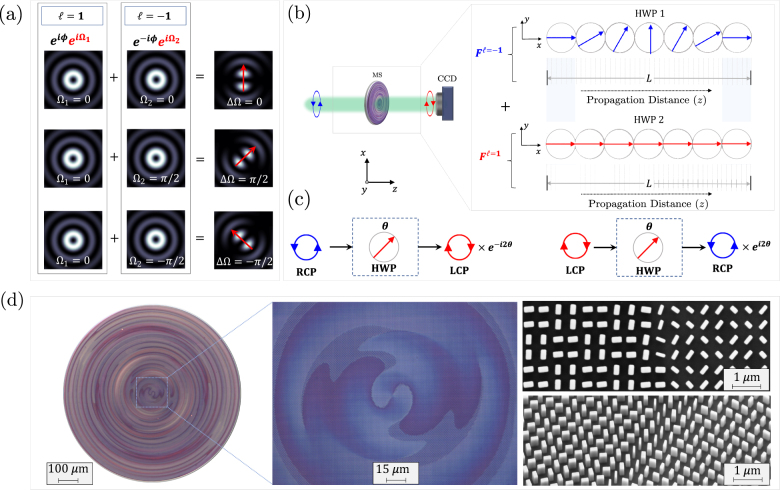
Optical vortex beams with spatially evolving Berry phase: design principle and implementation. (a) Superposition of two vortex modes with opposite helicity, *ℓ* = 1 and *ℓ* = −1, produces a petal structure which rotates depending on the relative phase shift (ΔΩ) between the vortex modes; ΔΩ = Ω_2_ − Ω_1_. (b) Schematic of our metasurface profile for detecting the Berry phase: two vortex modes with opposite helicity, *ψ* ^*ℓ* = −1^ and *ψ* ^*ℓ* = 1^, experience different HWP-like polarization transformations. The fast axis of HWP-1 rotates with propagation (blue arrows) whereas HWP-2 orientation is fixed (red arrows). Hence, only *ψ* ^*ℓ* = −1^ experiences a propagation-dependent polarization transformation. (c) A typical HWP would reverse the handedness of input RCP and LCP light at the output while imparting a Berry phase which depends on the orientation of its fast axis (*θ*). (d) Optical microscope and SEM images of the fabricated metasurface for the direct measurement of propagation-dependent Berry phase. The sample shows characteristic twisted patterns hinting at their function; converting a plane wave into two co-propagating vortex beams which result in rotating petal-like structure.

The propagation-dependent modification in the spin angular momentum gives rise to an additional spatially evolving Berry phase, as discussed in the previous section. The accumulated Berry phase can be directly measured using a wavefront sensor or by performing an interferometric measurement with a reference Gaussian beam at different propagation distances. Measurements of this nature, however, have their own challenges; the former is limited to low resolution whereas the latter is extremely sensitive to misalignment. Instead, here we perform an alternative interferometric measurement using a single metasurface without the need to include a reference arm beam or a wavefront sensor. To achieve this, we fabricated a metasurface that generates a superposition of two vortex beams of opposite helicity (*ℓ* = 1 and *ℓ* = −1). Vortex beams are a class of structured light that carries orbital angular momentum owing to their helical wavefront and on-axis phase singularity, where ℏ*ℓ* signifies the OAM per photon [60–65]. When two coherent OAM modes with opposite helicity are superimposed they interfere to produce a petal-like structure which in turn rotates clockwise (or counter clock wise) depending on the relative phase shift between the two OAM modes [66], see for e.g. Figure 4(a). The angular orientation of these petal structures provides a direct measure of the relative phase shift between its individual modes; simply by detecting the intensity profile.

More specifically, our metasurface implements superposition of two waveforms (*ψ* ^*ℓ* = −1^ + *ψ* ^*ℓ* = 1^). The polarization response **F** ^*ℓ*^ of *ψ* ^*ℓ* = −1^ is chosen to mimic an HWP which rotates its fast axis along the *z*-direction (i.e., akin to the response in Figure 3), whereas the polarization response of *ψ* ^*ℓ* = 1^ is set as an HWP whose fast axis is fixed. When illuminated by a plane wave, the metasurface will produce two co-propagating vortex modes of opposite helicity, *ℓ* = −1 and *ℓ* = 1, creating petal-like interference patterns like the ones in Figure 4(a). Since only one of the two waveforms (*ψ* ^*ℓ* = −1^) changes its polarization state with propagation, a relative Pancharatnam–Berry phase shift will arise between *ψ* ^*ℓ* = −1^ and *ψ* ^*ℓ* = 1^ which can be inferred from intensity measurements on a CCD. To demonstrate full control, we designed the polarization behavior of *ψ* ^*ℓ* = −1^ to mimic an HWP whose axis slowly rotates in one direction (counter clockwise, CCW) over one space region and then rotates back (clockwise, CW), as a function of propagation distance, as illustrated by the blue arrows in Figure 4(b). By design, we chose the reversal in this adiabatic rotation to occur at the plane *z* = 16 mm. Figure 4(c) shows how a conventional HWP responds to circularly polarized light; reversing its chirality at the output while imparting a geometric phase that is twice the angle of the fast-axis. This behavior is spin-dependent. Similarly, when illuminated by right handed circularly polarized (RCP) light, our metasurface produces co-propagating vortices with left hand circular polarization (LCP), reversing the input chirality as expected from an HWP, while rotating its polarization adiabatically with propagation. This transformation allows *ψ* ^*ℓ* = −1^ to accumulate a Berry phase factor besides its propagation phase. In contrast, *ψ* ^*ℓ* = 1^ only accumulates the usual propagation phase with no Berry phase. Therefore, any rotation in the resulting petal structure will serve as a direct observation of the Berry phase factor accumulated by *ψ* ^*ℓ* = −1^, given that the dynamic phase difference accumulated with propagation is cancelled out. A device that can generate this petal-like profile is shown in Figure 4(d) which exhibits optical micrographs and SEM images of a metasurface (924 μm in diameter).

**Figure 5:**
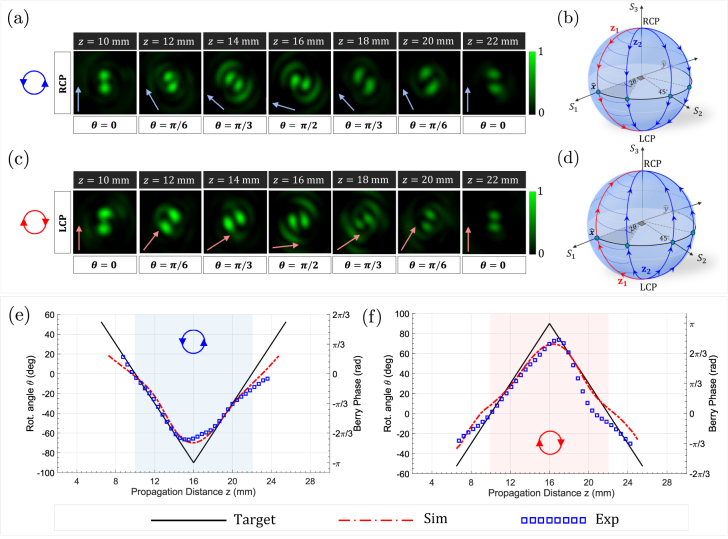
Direct measurement of spatially evolving Berry phase. (a and c) Measured transverse intensity profile of the metasurface in response to RCP (a) and LCP (c) input light. At each *z*-plane, the change in angular orientation of the petal structure suggests an additional Pancharatnam–Berry phase factor acquired by *ψ* ^*ℓ* = −1^, equal to (−2*σθ*) where *σ* = ±1 for RCP and LCP input light and *θ* is the fast axis orientation of HWP-1. The petals reverse their sense of rotation, consistent with changing *θ*. (b and d) The evolution in the polarization of *ψ* ^*ℓ* = −1^ (blue trajectory) and *ψ* ^*ℓ* = 1^ (red trajectory) are shown on the Poincaré sphere for RCP (b) and LCP light (d). (e) Measured and simulated orientation of the rotating petal structures in (a) as a function of the propagation distance, compared to the designed HWP response. The response is spin-dependent; input LCP light exhibits opposite behavior at the output as shown in (f). In both cases, the magnitude of the accumulated Berry phase is evaluated as twice the angular orientation of the petal structure (as well as twice the fast axis orientation of HWP-1, *θ*).

The measured intensity profile at the output of the metasurface is shown in Figure 5(a) in response to input RCP light. The arrows depict the orientation of the rotating petals and *θ* denotes the polarization response of *ψ* ^*ℓ* = −1^; namely the fast axis orientation of its HWP with respect to the horizontal axis (blue arrows in Figure 4(b)). Note how the petal structure rotates in the CCW direction then stops and reverses its sense of rotation to the CW direction, suggesting a variable phase shift between *ψ* ^*ℓ* = −1^ and *ψ* ^*ℓ* = 1^. When the same metasurface is illuminated by the orthogonal (LCP) polarization, the petal structure still rotates but the sense of rotation at each location is reversed, as shown in Figure 5(c). We attribute this rotation to the Pancharatnam–Berry phase accompanying the polarization transformation of *ψ* ^*ℓ* = −1^. To reconcile this, note that at each *z*-plane the polarization state of input RCP light becomes LCP at the output. The two co-propagating modes *ψ* ^*ℓ* = −1^ and *ψ* ^*ℓ* = 1^ undergo this polarization transformation via two different paths on the Poincaré sphere, as illustrated in Figure 5(b): (i) the blue paths (signified by different longitudes on the Poincaré sphere) are the trajectories traversed by *ψ* ^*ℓ* = −1^ and (ii) the red path is the fixed trajectory of *ψ* ^*ℓ* = 1^. The *z* = 0 plane lies at the focus of a 4*f* optical system after the metasurface (see Figure 2(e)) where both the red and blue trajectories coincide. As the waveform propagates, the polarization of *ψ* ^*ℓ* = −1^ and *ψ* ^*ℓ* = 1^ remain the same but the solid angle between the red and blue trajectories (given by 4*θ*) increases adiabatically. In this case, a variable Berry phase factor is accumulated, only by *ψ* ^*ℓ* = −1^, and hence the petal structure is rotated. After a longer propagation distance, precisely at *z* = 16 mm (as per our design), these dynamics are reversed, the solid angle between the blue and red trajectories progressively decreases, and the petal structure eventually retains its initial orientation. Evidently, this response is spin-dependent; it reverses its topology depending on the chirality of incident light. In both cases, the additional Berry phase factor can be geometrically evaluated as half the solid angle between the red path and the blue path on the Poincaré spheres of Figure 5(b) and (d), which illustrate both the case of input RCP (top) and LCP (bottom).

To quantify the accumulated phase, we measured the angular orientation of the rotating petal structure at each *z*-plane under the two input polarizations RCP and LCP. We achieved this by tracking the petal orientation using a robust image processing algorithm which tracks the center of mass of each lobe and estimates their tilt angle. Figure 5(e) depicts the result of this analysis. Under RCP illumination, the petal structure rotates in the CCW direction, acquiring a negative Berry phase factor which is accumulated at a linear rate along the optical path. At *z* = 16 mm, the rotating petal stops then reverses its sense of rotation as well as the slope by which the Berry phase is accumulated. This picture is mirrored for the case of input LCP light, as shown in Figure 5(f). For a petal structure composed of vortex modes ±*ℓ*, the acquired Berry phase value is equal to −2*σ*|*ℓ*|*θ* as depicted on the right axis for the plots, where *θ* denotes the fast axis orientation of HWP-1 (which also coincides with the magnitude of the petal’s angular orientation) and *σ* is the polarization handedness. As the beam reverses its sense of rotation, at *z* = 16 mm, it experiences angular acceleration/deceleration—a behavior that is captured by the flat valley and peak in the measured and simulated results of Figure 5(e) and (f), respectively, but not seen in their target response (which neglects these effects). Note that the measured orientation deviates from the simulation towards the edges of the region of interest (i.e., at *z* = 9 and 22 mm). One possible reason for this discrepancy, besides fabrication tolerances, is that we are dealing with apertured Bessel beams in which a small contribution of the beam’s angular momentum (stored in its outer most rings) is truncated and thus does not contribute to the propagation dynamics. This perturbs the angular rotation, especially at the edges of the propagation range, where the contributions from the beam’s peripherals become more significant. One can mitigate this discrepancy by extending the aperture size (metasurface diameter) and/or by including more Bessel terms in Eq. (1) to better approximate the target behavior.

The phase gradient along *z* translates to an effective momentum which perturbs the *k*_*z*_ component of the wavevector, thus modifying the beam’s size (see also Figure 3(g)–(i) and associated discussion). Owing to the energy-momentum conservation, such perturbation manifests as a shift in the transverse spatial frequencies of the output waveform. Figure 5(a) confirms this behavior; under RCP illumination the transverse beam size is slightly larger over the space region *z* > 16 mm (where the Berry phase gradient is positive). This effect is reversed under LCP illumination, where the beam’s dimensions are larger over the region *z* < 16 mm. Therefore, judicious design of the polarization transformation provides a new degree-of-freedom for tailoring the phase response along the optical path. It is worth noting that an analogous effect has been previously observed in time-domain; a coherent Gaussian beam experiencing an adiabatic evolution in its polarization state as a function of time also accumulates a time-dependent linear Berry phase which translates to a temporal frequency shift [67].

## 4 Discussion and outlook

To the best of our knowledge, we reported the first direct observation of a longitudinally evolving Pancharatnam–Berry phase under free-space propagation. We examined a new class of polarization meta-optics which implements a superposition of Bessel beams with different cone angles, each weighted by a different Jones matrix, allowing the spin angular momentum (polarization state) of the ensemble to be tailored at-will along the optical path. These polarization transformations are accompanied by a propagation-dependent geometric phase factor which, unlike the monotonically accumulated propagation phase, can be designed on demand along the direction of propagation. For e.g., in Figure 5(e) we presented a scenario in which the phase gradient (with respect to *z*) can be negative over one space region and positive over another.

Here, we paid particular attention to Pancharatnam–Berry phase accompanying the polarization transformation of Bessel beams. This has been done by designing the response of our metasurface to mimic an HWP whose fast axis rotates by an angle of *θ* along the optical path, giving rise to a phase factor of −2*σθ*. Besides the HWP profile, our approach can realize other polarization responses and trajectories on the Poincaré sphere. To demonstrate this, we considered another device that mimics the function of a longitudinally variable quarter-wave plate thus modifying the chirality—i.e., the spin angular momentum—of incident light along the optical path. More details on this device can be found in Supplementary Figure 2. More generally, one can, in principle, implement a metasurface that performs a propagation-dependent rotation of the entire spatial coordinate system (not only the polarization state) by designing the longitudinal response **F** ^*ℓ*^ to mimic the 2-by-2 rotation matrix *R*(*θ*(*z*)). In this case, the accumulated Berry phase becomes a function of the total angular momentum and is given by −(*σ* + *ℓ*)*θ*, as predicted by Bliokh for the 2D case [38]. A metasurface profile of this nature, composed of an asymmetric 2-by-2 matrix, however, cannot be implemented using the single layer metasurface deployed in this work as it requires elliptical form birefringence for the metasurface unit cells. This requirement can be achieved by using cascaded or bilayer metasurfaces. We reserve the demonstration of these higher order Berry phases [39, 40] and their analysis to other future work.

Controlling the spin angular momentum and Berry phase, as demonstrated in this work, can inspire new directions in science and technology. It redefines basic rules of wave propagation and points towards a new route to tailoring the phase gradient along the optical path. This can have significant impact on nonlinear interactions and can enable more compact cavity designs and photonic devices [68]. Given our choice of Bessel functions as the OAM modes, our devices generate pencil-like beams characterized by a nondiffracting and self-healing behavior [56] which are also desirable in micromanipulation and free space optical communications. Furthermore, our work enables topologically complex states of light which in turn can lead to many new phenomena in quantum and classical optics [69]. Besides their potential application in light–matter interaction and free-space communications, the compact form of our devices enables their integration in laser cavities, defining new rules for the phase matching condition, and generating new topologically complex combinations of SAM and OAM states of light at the source [70–72]. Lastly, the multidisciplinary nature of angular momentum across different fields may inspire related research efforts in the areas of microfluidics, acoustics, and electron beams, to name a few.

## Supplementary Material

Supplementary Material Details
