# Supplementary material for: Introducing Berry phase gradients along the optical path via propagation-dependent polarization transformations
Source: Nanophotonics. 2021 Nov 19;11(4):713–25. doi: 10.1515/nanoph-2021-0560 (PMC11501737; doi:10.1515/nanoph-2021-0560)
Supplement: Supplementary file 1 — Supplementary Material Details [file j_nanoph-2021-0560_suppl.pdf]

Supplementary Information

Ahmed H. Dorrah, Michele Tamagnone, Noah A. Rubin, Aun Zaidi, and Federico Capasso

Introducing Berry phase gradients along the optical path via propagation-dependent polarization transformations

1 Metasurface Implementation

1.1 Evaluating the Target Profile

The target amplitude-phase-polarization profile that we wish to implement using a metasurface is given by Eq. (1) in the main text; a superposition of Bessel modes, equally separated in k_z space, and each multiplied by a different 2-by-2 Jones matrix as follows

$$\tilde{\psi}^{\ell}(\rho,\phi,z,t) = e^{-i\omega t}\sum_{m=-N}^{N}\tilde{\mathbf{A}}^{\ell,m} J_{\ell}(k_{\rho}^{\ell,m}\rho)e^{i\ell\phi}e^{ik_z^{\ell,m}z}. \tag{S1}$$

The coefficients $\tilde{\mathbf{A}}^{\ell,m}$ in this series are obtained by solving

$$\tilde{\mathbf{A}}^{\ell,m} = \frac{1}{L}\int_0^L \tilde{\mathbf{F}}^{\ell}(z)e^{-i\frac{2\pi}{L}mz}\,\mathrm{d}z. \tag{S2}$$

To evaluate these coefficients, we first define our desired polarization response, $\tilde{\mathbf{F}}(z)$. Here, we are interested in meta-optics which mimic a retarder (HWP) that rotates its fast axis along the optical path. To realize this response, we set $\tilde{\mathbf{F}}(z)$, as the 2-by-2 Jones matrix given by

$$\tilde{\mathbf{J}}_{\mathrm{WP}}(z) = \begin{bmatrix}\cos(\Theta/2)+i\sin(\Theta/2)\cos(2\theta_z) & i\sin(\Theta/2)\sin(2\theta_z)\\ i\sin(\Theta/2)\sin(2\theta_z) & \cos(\Theta/2)-i\sin(\Theta/2)\cos(2\theta_z)\end{bmatrix}, \tag{S3}$$

where Θ denotes the retardance, i.e., relative phase shift between the eigen polarizations of the wave plate (e.g., $\Theta=\pi,\pi/2$ for a half- and a quarter-wave plate, respectively) and we have denoted the propagation dependent angular orientation, $\theta(z)$, as θ_z for clarity. Here, θ is a predetermined function of z. By substituting this expression in Eq. (S2), we obtain the coefficients $\tilde{\mathbf{A}}^{\ell,m}$. Afterwards we evaluate the ensemble $\tilde{\psi}^{\ell}(\rho,\phi,z,t)$

Ahmed H. Dorrah, Noah A. Rubin, Aun Zaidi, Federico Capasso, Harvard John A. Paulson School of Engineering and Applied Sciences, Harvard University, Cambridge, Massachusetts 02138, USA.

Michele Tamagnone, Harvard John A. Paulson School of Engineering and Applied Sciences, Harvard University, Cambridge, Massachusetts 02138, USA and Fondazione Istituto Italiano di Tecnologia, Genova, Italy.

Corresponding author(s): Ahmed H. Dorrah (dorrah@seas.harvard.edu) and Federico Capasso (capasso@seas.harvard.edu).

from Eq. (S1) at the plane $z = 0$ to obtain the target profile. This represents the transverse profile that we wish to implement; a distribution of spatially varying Jones matrices that will transform an incident plane-wave into a pencil-like beam that changes its polarization as a function of propagation distance.

1.2 Design Considerations and Beam Dimensions

To provide an example, consider the device reported in Figs. (4) and (5) of the main text which produced a rotating petal-like structure. For this design, we set $N = 6$ which yields 13 Bessel modes for each OAM series ψ^ℓ. Further, the longitudinal wavevectors $k_z^{(0)}$ were centered at a value of 0.999925 ω/c with a separation of $2\pi/L$ in k_z-space, where $L = 50$ mm. This choice of N and the wave vectors ensures operation in the paraxial regime. Our choice of parameters is also intricately related to the generated beam's range and aperture size (diameter) of the plates. Essentially, a larger plate can generate vortex modes over a longer range—a relation that is precisely governed by the geometric argument of axicons [1]. In general, the minimum aperture diameter needed to generate a non-diffracting Bessel beam over a range L is given by

$$D \geq 2L\sqrt{\left(\frac{k_0}{k_z^{m=-N}}\right)^2 - 1}, \tag{S4}$$

where $k_0 = \omega_0/c$, and $k_z^{m=-N}$ is the smallest longitudinal wavenumber in the sum of Eq. (S1). Equation (S4) is valid for Bessel beams of 0-th order in which the energy is localized over a central spot. For $\ell \geq 1$ where the energy of the beam is distributed over a ring, i.e. OAM beams, the aperture size shall be larger than D plus the vortex beam's diameter.

Longitudinal Resolution

Besides scaling the transverse dimensions of the beam and its propagation range, the adiabaticity of polarization evolution can be designed. For instance, in Fig. 3(b) of the main text, we detected polarization rotation with an increments of 6° per 100 μm along the z-axis. This is a design parameter that can be controlled. To reconcile this, recall that the longitudinally variable response of our devices relies on spatial polarization beating in the envelope of co-propagating Bessel beams owing to their different k-vectors. The separation between the longitudinal wavenumbers ($2\pi/L$) determines the periodic modulation of the output waveform, and is given by the distance L. Additionally, the absolute value of the wavenumbers (spatial frequencies) governs how abruptly the envelope can be modulated within the interval L. To modulate the polarization over very short distances along z-direction (with high resolution), the metasurface should be able to implement Bessel functions with a wide range of small and large spatial frequencies, akin to a Fourier series. Various profiles of longitudinally varying polarization can be realized provided that the spatial harmonics in the sum are sufficient to synthesize the ensemble. To achieve this, the metasurface should be able to sample the transverse profile of the highest-frequency Bessel function in the sum given by Eq. (S1). Since our metasurfaces can sample a waveform with sub-wavelength (420 nm) resolution, it follows that Bessel functions with large spatial frequency, up to $k_r \leq \frac{1}{2\times 420\text{nm}}$, can be supported without violating Nyquist's sampling criterion. In our devices, the largest transverse wavenumber was chosen as $k_r \approx 5 \times 10^5$ m^{-1} which is an order of magnitude smaller than the limit imposed by our metasurface resolution.

1.3 Dual Matrix Holography

As stated earlier, solving for $\tilde{\psi}^{\ell}(\rho, \phi, z = 0)$ yields our target transverse profile which can be described as a spatially varying distribution of Jones matrices. As shown below, our metasurface unit cells can be regarded as retarders which control the phase and polarization of the incident beam, but with no amplitude control. Our target profile $\tilde{\psi}^{\ell}(\rho, \phi, z = 0)$, however, requires amplitude modulation. To achieve this, we used a recently developed holography technique, dubbed dual matrix holography [2], which allows us to modulate both the amplitude and phase of an incoming wavefront using a phase-only (unitary) metasurface platform. The underlying principle is the matrix-analog of detour phase and double-phase holograms [3] in which two adjacent pixels can introduce a relative phase delay on incident light to modulate its amplitude at the far-field via interference. Similarly, we decompose each pixel of $\tilde{\psi}^{\ell}(\rho, \phi, z = 0)$ into two adjacent phase-only pixels, compatible with our meta-atoms, to realize full phase-amplitude-polarization control away from the metasurface. This is possible since any hermitian matrix (i.e., with non unity eigen vectors) can be decomposed into the sum of two unitary matrices. Complex amplitude modulation in this way, however, is achieved on the expense of higher diffraction orders which act as loss channels for our device, yielding an overall transmission efficiency of $\sim 20\%$ or less.

1.4 Mapping the Design to Metasurface Unit Cells

After converting the target profile, $\tilde{\psi}^{\ell}(\rho, \phi, z = 0)$, to unitary Jones matrices via dual matrix holography, the output profile will be compatible with our metasurface platform. We adopted a metasurface configuration made of rectangular dielectric nanofins of high index contrast. The nanofins were made of Titanium Dioxide (TiO_2) with a fixed height of 600 nm on top of a glass substrate, as depicted in Fig. S1(a). These unit cells support two propagating modes which due to anisotropy experience different indices (phase delays). Each nanofin can be thought of as a unitary waveplate given by the 2-by-2 Jones matrix

$$\tilde{J}(x, y) = \mathbf{R}(-\Phi(x, y)) \begin{bmatrix} e^{i\Theta_x(x,y)} & 0 \\ 0 & e^{i\Theta_y(x,y)} \end{bmatrix} \mathbf{R}(\Phi(x, y)). \quad \text{(S5)}$$

The phase retardances (Θ_x and Θ_y) in Eq. (S5) can be tuned by varying the dimensions of the nanofins in the transverse (i.e., x-y) plane, whereas the local rotational angle Φ can be adjusted by changing the nanofin's angular orientation about the longitudinal axis, z, allowing us to implement the target profile, point-by-point. Figs. S1(b) and (d) show the transmission and phase response encoded by each nanofin on an x-polarized incident plane wave, as a function of the nanofin dimensions. These results were numerically evaluated using FDTD. The phase shift was obtained by probing the center of a monitor located above the structure in the far field, whereas the power transmission is the total power passing through a monitor above the structure relative to the input power. We selected nanofins of uniform transmission to avoid operation near resonances which suffer from narrow operation bandwidth and low fabrication tolerance. For this reason, our metasurface unit cells do not alter the amplitude of incident light. The average transmission efficiency of the the individual nanofins is $\sim 70\%$. Fig. S1(c) depicts the complex transmission coefficient imparted on x- polarized light, $t = t_x e^{i\Theta_x}$. Each dot corresponds to one simulated geometry. The radius from the origin corresponds to t_x and the angle of the dot relative to the x axis is Θ_x. Our metasurface library can provide

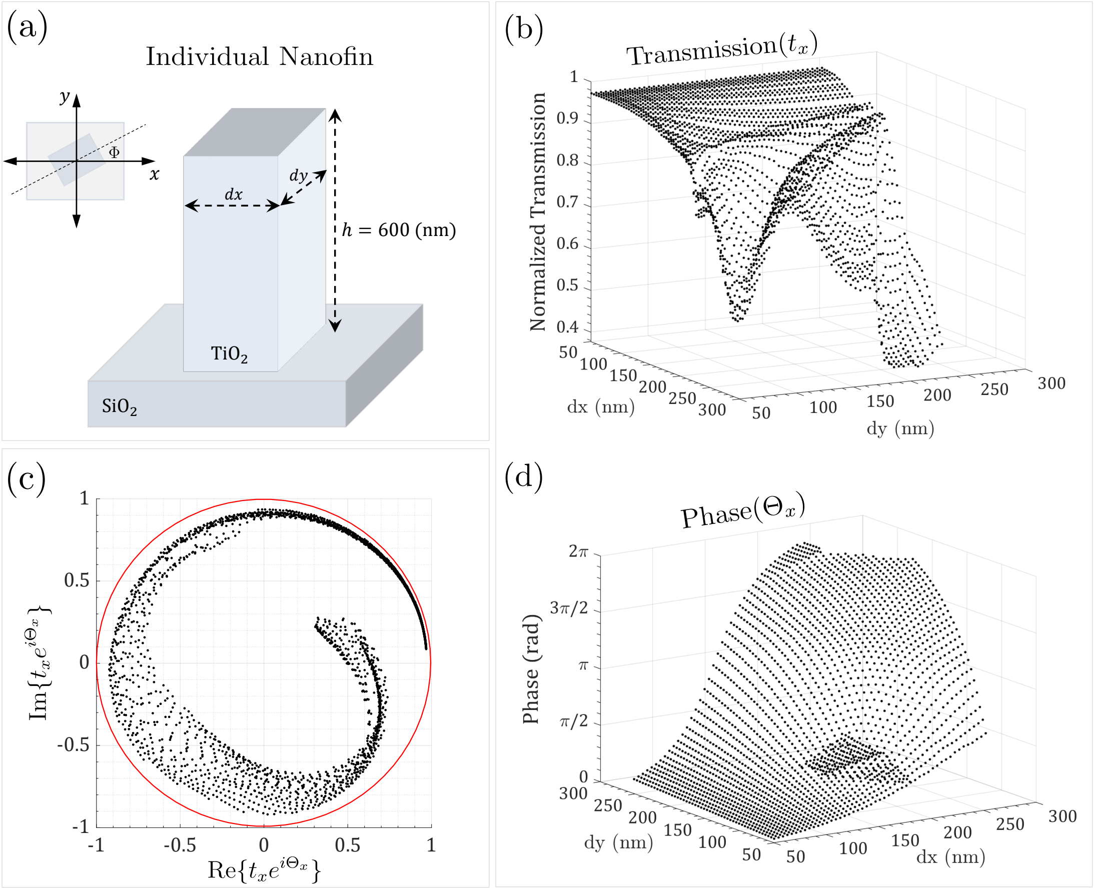

Fig. S1: Metasurface Design. **(a)** Schematic of the metasurface unit cell composed of a rectangular nanofin made of Titanium Dioxde (TiO_2) with a fixed height of 600 nm on top of a glass substrate[4]. **(b)** Normalized power transmission of the nanofins as a function of d_x and d_y, for input x-polarization. **(c)** The electric field amplitude transmission $t_x e^{i\Theta_x}$ plotted on the complex plane for each of the 2500 individual geometries (black dots). The red circle is the unit circle. **(d)** Phase response of the nanofins (in Radians) as a function of the transverse dimensions, d_x and d_y, for incident x-polarization. Each black dot corresponds to a specific nanofin geometry. From the symmetry, the nanofin response for input y-polarization is readily obtained by swapping x and y in (b) and (d).

almost full $[0, 2\pi]$ phase coverage with quasi uniform transmission over large number of geometries. A similar response applies to y-polarized light, from symmetry. Each nanofin can thus impart two independent phase profiles on light polarized along its major and minor axes. Furthermore, the rotational degree-of-freedom of each nanofin, combined with its linear form birefringence, can be exploited to manipulate the incident polarization, point-by-point, as described by Eq. (S5). In mapping our profiles to metasurface unit cells, we assume a 60-level phase discretization for Θ_x and Θ_y, and a continuous range for Φ.

The three-step selection process of each individual nanofin of the designed metasurface is summarized as follows: a) The complex-valued errors ϵ_x and ϵ_y are first evaluated from $\epsilon_x = |t_{\text{avg}} e^{i\Theta_{x,\text{desired}}} - t_{\text{simulated}} e^{i\Theta_{x,\text{simulated}}}|$ and $\epsilon_y = |t_{\text{avg}} e^{i\Theta_{y,\text{desired}}} - t_{\text{simulated}} e^{i\Theta_{y,\text{simulated}}}|$, for all possible nano pillar configurations. b) For each geometry, the maximum error $\epsilon_{\max} = \max(\epsilon_x, \epsilon_y)$ is determined, and finally c) the configuration that minimizes $\epsilon_{\max}$ is selected. This process, which is now standard in the metasurface literature, is further described in Ref [5].

1.5 Device Fabrication

A positive tone electron beam resist was spin coated on top of a fused Silica substrate, ultimately defining the nanofins height. The resist was first baked then exposed using electron beam lithography (with accelerating voltage of 125 kV), writing the desired nanofin pattern. The exposed pattern was developed by submerging the sample in o-xylene for 60 sec, creating the desired geometry of the individual nanofins. Afterwards, atomic layer deposition process was used to depost TiO_2, conformally filling the developed pattern. The excess TiO_2 layer covering the device was etched away to the original height of the resist via reactive ion etching (RIE). Finally, the resist was chemically removed leaving the TiO_2 nanofins surrounded by air. More details on our fabrication procedure can be found in Ref. [4].

1.6 Achromatic Response

Throughout this work we utilized a simple meta-atom scheme which consists of rectangular nanofins with a center-to-center separation of 420 nm. Our library of meta-atoms was optimized for the 532 nm wavelength. In principle, one can achieve a broadband response over a wide range of wavelengths using more complicated metasurface configurations which make use of coupling between neighboring unit cells, or use cascaded metasurfaces, and guided mode resonances for multiwavelength operations. A comprehensive review on dispersion-engineered metasurfaces can be found in Ref. [6]. This was not the focus of the current study but can be the subject of future work.

2 Polarization Characterization

We performed Stokes polarimetry to measure the polarization state at the input and output of the devices, thus retrieving the four-component polarization Stokes vector, $\vec{S} = (S_0, S_1, S_2, S_3)^{\mathbf{T}}$. Here, $\vec{S}$ quantifies the shape and orientation of the polarization ellipse at each point in space addition to the beam's intensity and degree of polarization [7]. The Stokes parameters were obtained by rotating a polarizer (Pol) and a quarter-wave plate (QWP) before the CCD to analyze for the polarization states: 0°, 45°, 90°, and right hand circular polarization (RCP). We denote the corresponding intensities as $I_{0^\circ}, I_{45^\circ}, I_{90^\circ}$, and I_{RCP}, respectively. At each z-plane, the four Stokes parameters were obtained as follows: $S_0 = I_{0^\circ} + I_{90^\circ}$, $S_1 = I_{0^\circ} - I_{90^\circ}$, $S_2 = 2(I_{45^\circ}) - (I_{0^\circ} + I_{90^\circ})$, and $S_3 = 2(I_{\mathrm{RCP}}) - (I_{0^\circ} + I_{90^\circ})$.

3 Berry phase gradients in propagation-dependent QWP

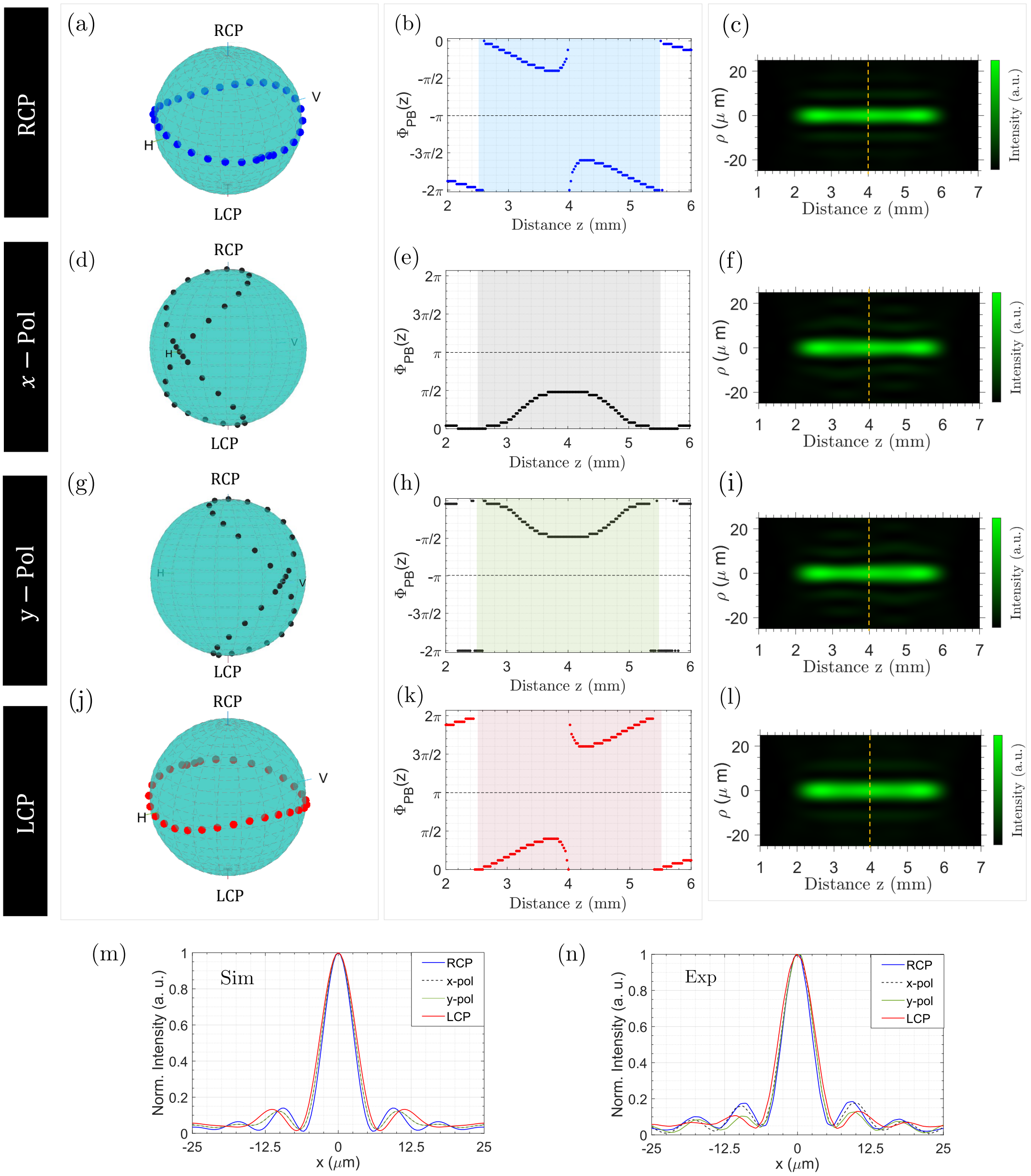

Fig. S2: Spatial evolution of polarization and Pancharatnam-Berry phase in longitudinally varying quarter-wave plate (QWP). **(a)** Trajectory of the polarization state, visualized on the Poincaré sphere, when the meta-optic is illuminated by RCP input light. Here, the meta-optic mimics a QWP which rotates its fast axis orientation by an angle θ, which varies as function of z, from 0° to 180° with respect to the x-axis. **(b)** Geometric phase associated with the propagation-dependent polarization transformation traversed in **(a)**. **(c)** Longitudinal intensity profile at the output of the meta-optic. The dashed lines mark cross sectional cuts at $z = 4$ mm. Similar results are plotted for input x-polarization **(d-f)**, y-polarization **(g-i)**, and LCP **(j-l)**. The corresponding simulated and measured transverse profiles (1D cuts) are depicted in **(m-n)** and show a slight variation in the beam's size, depending on the input polarization, and implying a slight shift in the spatial frequency (transverse and longitudinal wavevectors) of the beam.

References

[1] McGloin, D. & Dholakia, K. Bessel beams: Diffraction in a new light. *Contemporary Physics* **46**, 15–28 (2005).

[2] Dorrah, A. H., Rubin, N. A., Zaidi, A., Tamagnone, M. & Capasso, F. Metasurface optics for on-demand polarization transformations along the optical path. *Nature Photonics* **15**, 287–296 (2021).

[3] Hsueh, C. K. & Sawchuk, A. A. Computer-generated double-phase holograms. *Appl. Opt.* **17**, 3874–3883 (1978).

[4] Devlin, R. C., Khorasaninejad, M., Chen, W. T., Oh, J. & Capasso, F. Broadband high-efficiency dielectric metasurfaces for the visible spectrum. *Proceedings of the National Academy of Sciences* **113**, 10473–10478 (2016).

[5] Balthasar Mueller, J. P., Rubin, N. A., Devlin, R. C., Groever, B. & Capasso, F. Metasurface polarization optics: Independent phase control of arbitrary orthogonal states of polarization. *Phys. Rev. Lett.* **118**, 113901 (2017).

[6] Chen, W. T., Zhu, A. Y. & Capasso, F. Flat optics with dispersion-engineered metasurfaces. *Nature Reviews Materials* **5**, 604–620 (2020).

[7] Chipman, R. A., Lam, W. S. T. & Young, G. *Polarized light and optical systems.* (Boca Raton, FL : CRC Press, 2019).
